# Clinical Significance of Manuka and Medical-Grade Honey for Antibiotic-Resistant Infections: A Systematic Review

**DOI:** 10.3390/antibiotics9110766

**Published:** 2020-10-31

**Authors:** Victoria C. Nolan, James Harrison, John E. E. Wright, Jonathan A. G. Cox

**Affiliations:** 1 College of Health and Life Sciences, Aston University, Aston Triangle, Birmingham B4 7ET, UK; 180208508@aston.ac.uk (V.C.N.); j.harrison11@aston.ac.uk (J.H.); 2 Department of Intensive Care Medicine, Great Western Hospital NHS Foundation Trust, Swindon SN3 6BB, UK; john.wright14@nhs.net

**Keywords:** honey, Manuka honey, medical-grade honey, antibiotic resistance

## Abstract

Antimicrobial resistance is an ever-increasing global issue that has the potential to overtake cancer as the leading cause of death worldwide by 2050. With the passing of the “golden age” of antibiotic discovery, identifying alternative treatments to commonly used antimicrobials is more important than ever. Honey has been used as a topical wound treatment for millennia and more recently has been formulated into a series of medical-grade honeys for use primarily for wound and burn treatment. In this systematic review, we examined the effectiveness of differing honeys as an antimicrobial treatment against a variety of multidrug-resistant (MDR) bacterial species. We analysed 16 original research articles that included a total of 18 different types of honey against 32 different bacterial species, including numerous MDR strains. We identified that Surgihoney was the most effective honey, displaying minimum inhibitory concentrations as low as 0.1% (w/v); however, all honeys reviewed showed a high efficacy against most bacterial species analysed. Importantly, the MDR status of each bacterial strain had no impact on the susceptibility of the organism to honey. Hence, the use of honey as an antimicrobial therapy should be considered as an alternative approach for the treatment of antibiotic-resistant infections.

## 1. Introduction

The unique antimicrobial and anti-inflammatory properties of honey have been established for millennia [1]. It has been used to treat a plethora of ailments throughout history, from gout to pain relief [2]. Honey research has been focused on identifying the unique properties that give rise to its medicinal uses as well as establishing efficacy and exploring potential uses [3]. Honey exhibits a spectrum of antimicrobial activity, inhibiting a variety of microorganisms [4]. It has been well established that the main components attributed to honey’s ability to inhibit microorganisms are hydrogen peroxide, polyphenolic compounds and bee defensin-1, which are all present within honey at varying levels [5]. Other factors that contribute are a high osmolarity, high sugar content and low pH [6,7]. These components vary largely between honeys and are dependent on the floral origin, geographical location and honeybee physiology [8]. Therefore, every honey has the possibility to inhibit a variety of microorganisms, which has been observed in several studies with honeys from differing geographical location and botanical origin [3,9,10,11,12,13,14,15,16,17,18]. One particular honey that highlights the influence of these factors and that attracts the most attention is Manuka honey. Derived from the *Leptospermum* spp., or Manuka tree, it has been identified for its increased antimicrobial activity against a range of microorganisms [19]. The increased activity is attributed to the presence of methylglyoxal, not present in other honeys [20]. This is due to dihydroxyacetone, identified only in the flower nectar of *Leptospermum* spp., and is a precursor for methylglyoxal [21]. Through honey maturation, the dihydroxyacetone is converted non-enzymatically to methylglyoxal, resulting in its presence within Manuka-based honey [22]. Manuka honey still contains the other main components that give rise to honey’s antimicrobial activity; however, the presence of methylglyoxal result in increased activity. The observations of Manuka honey’s potent activity led to the development of medical-grade honey, which now has a variety of uses within a clinical setting. The main uses for medical-grade honey are as a topical ointment and honey-laced dressings in the treatment of surface wounds and burns [23]. These both work to promote wound healing but more importantly prevent and treat microbial infection, especially those caused by multidrug-resistant microorganisms [24]. 

The rise of antimicrobial resistance and the continued prevalence of multidrug-resistant (MDR) microorganisms has become one of the largest threats to face modern medicine [25]. If left unchallenged, antimicrobial resistance mortality could reach 10 million deaths per year by 2050 [26]. Some of the clinically significant MDR pathogens include *Staphylococcus aureus*, methicillin-resistant *S. aureus* (MRSA), *Pseudomonas aeruginosa*, *Escherichia coli*, extended-spectrum β-lactamase-producing (ESBL) *E. coli* and vancomycin-resistant enterococci (VRE). These pathogens can cause invasive disease, are highly transmissible and are associated with increased mortality [27]. The multidrug-resistant nature of these organisms makes them difficult to treat, and with limited antibiotics on the horizon, new and novel treatments are urgently required to combat infections. The development of medical-grade honey has provided a promising future for wound and burn treatment, with improved healing and reduced scarring among some of the benefits to honey treatment [28,29,30]. However, there could be more avenues to be explored for honey as a therapy, due to its efficacy against MDR organisms, which is not largely discussed. This systematic review explores the efficacy of Manuka honey and medical-grade honeys against a variety of drug-susceptible and drug-resistant bacterial pathogens, to determine if honey should be further explored, utilised and implemented as a treatment. 

## 2. Results

A large variety Manuka honey and medical-grade honeys were used in this review (Table 1). The honeys were tested against a variety of drug-susceptible and drug-resistant bacterial pathogens, all exhibiting a range of inhibition. In total, 8 Manuka honeys and 10 medical-grade honeys were used. 

### 2.1. The Impact of Honey on Staphylococcus aureus

**Figure 1:**
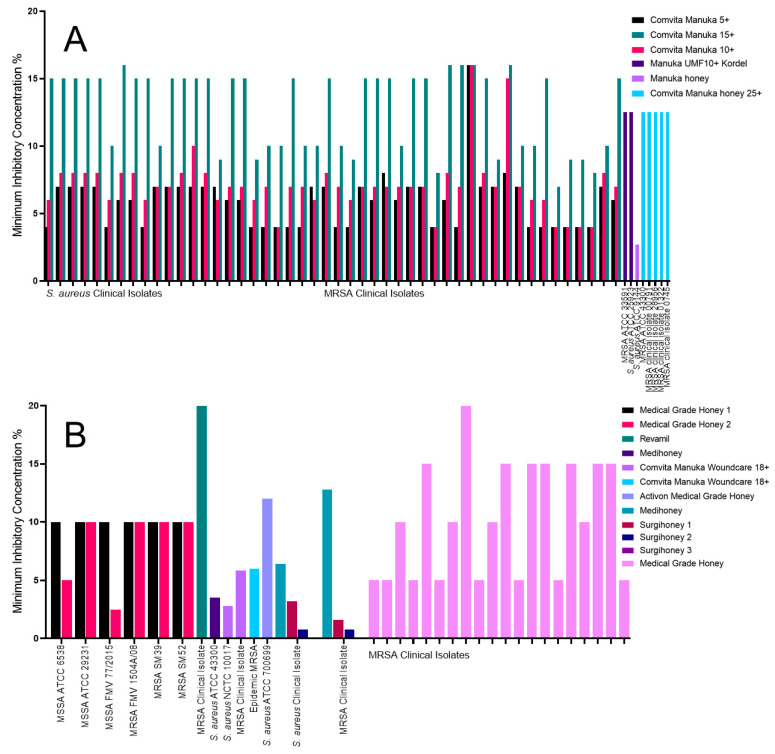
MICs of the *S. aureus* isolates tested against a variety of Manuka and medical-grade honeys. (**A**) All the Manuka honey MIC data used in this review, identifying Manuka honey and Comvita Manuka honey UMF5+ as the most effective; and (**B**) all the medical-grade honey MIC data used in this review, identifying all three Surgihoneys as the most effective.

A range of inhibition can be observed for both Manuka honey and medical-grade honey against a variety of *Staphylococcus aureus* isolates, with all MICs being 20% or less [31,32,33,35,37,38,39,40,41,42,45,46]. For Manuka honey, the Comvita Manuka 5+ exhibited the lowest MIC for almost all Manuka honey types tested against *S. aureus* (Figure 1A). This was also the lowest MIC for almost all of the clinical isolates tested by Girma et al. [31], in the range of <5% to 7% (w/v), regardless of methicillin resistance. The Comvita Manuka 10+ exhibited a similar effect with MICs ranging from <5% to 8%. The Comvita Manuka 15+ was the least effective honey tested, ranging from 8% to >15%. One MRSA clinical isolate was unaffected at the concentrations tested by all three of the Comvita Manuka honeys [31]. The ungraded Manuka honey was the only other honey to have such a low MIC at 2.7% (v/v) when tested against *S. aureus* ATCC 9144 [33]. The highest MIC observed was Comvita Manuka 15+, with MICs of 15% (w/v) or higher [31]. The Manuka UMF10+ Kordel (w/v) and Comvita Manuka 25+ (v/v) both inhibited all the tested *S. aureus* isolates at 12.5% [32,35]. The minimum bactericidal concentration (MBC) was also reported for both of these honeys, with Manuka UMF10+ Kordel having an MBC of 25% (w/v) and Comvita Manuka 25+ having an MBC of 12.5% (v/v), the same as the MIC. 

The medical-grade honeys had a greater variation in MIC than the Manuka honeys (Figure 1B). The medical-grade honey tested against a variety of MRSA clinical isolates inhibited at a range of 5% to 20%, a variation that was not observed for any of the Manuka honeys [46]. The Medihoney MIC for *S. aureus* ATCC 43300 was 3.5% and the MBC was 9.5% [40]; the same isolate was used against the Comvita Manuka 25+, which showed an MIC and MBC of 12.5% (v/v) (Figure 1B) [35]. The Medihoney was also used in a different study against a *S. aureus* clinical isolate and an MRSA clinical isolate, with MICs of 6.4% (w/v) and 12.8% (w/v), respectively, and MBCs of >25.6% [39]. The lowest MIC observed for the medical-grade honeys was for Comvita Manuka Woundcare 18+ with an MIC of 2.8% (w/v) against *S. aureus* NCTC 10017 [42]. The same honey against MRSA and epidemic MRSA had MICs of 5.83% (w/v) and 6% (w/v), respectively [41,42]. The medical-grade honeys 1 and 2 had some variation in inhibition. Regardless of the isolate tested, medical-grade honey 1 had an MIC of 10% (w/v) and for all but one isolate had an MBC of 10% (w/v). Whereas, medical-grade honey 2 had an MIC range of 2.5% (w/v) to 10% (w/v), and an MBC range of 5% (w/v) to 40% (w/v), depending on the isolate [37]. Activon medical-grade honey had an MIC of 12% (w/v) against *S. aureus* ATCC 700699 [45]. Revamil honey had the highest MIC at 20% (v/v) against an MRSA clinical isolate [38]. 

### 2.2. The Impact of Honey on Pseudomonas aeruginosa

**Figure 2:**
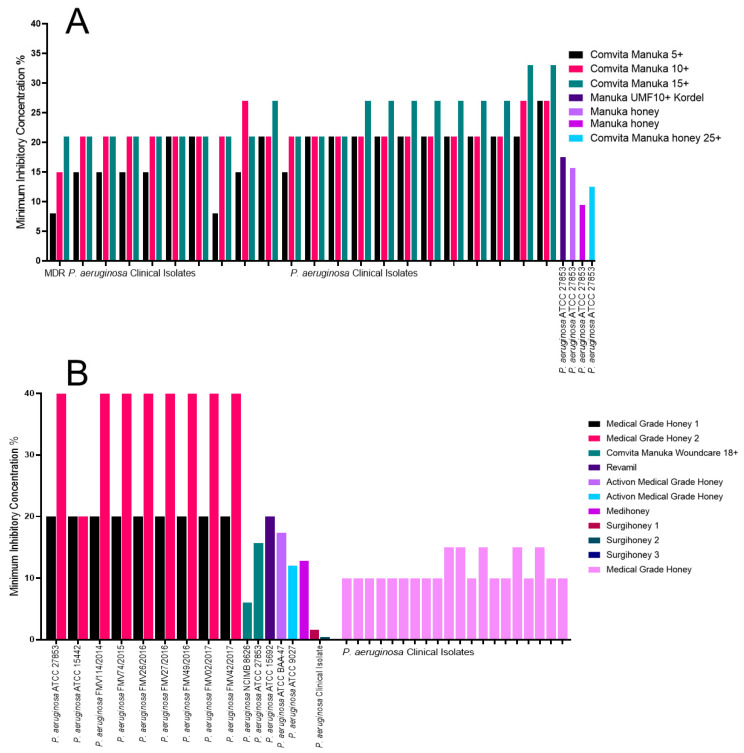
MICs of the *P. aeruginosa* isolates tested against a variety of Manuka and medical-grade honey. (**A**) All the Manuka honey MIC data used in this review, identifying the ungraded Manuka honey and Comvita Manuka UMF5+ as the most effective; and (**B**) all the medical-grade honey MIC data used in this review, identifying the three Surgihoneys as the most effective.

Variation in inhibition was observed for Manuka honey and the medical-grade honeys against *Pseudomonas aeruginosa* and clinical isolates [31,32,33,34,35,37,38,39,41,42,44,45,46]. The three Manuka honeys, Comvita Manuka 5+, 10+ and 15+, tested against *P. aeruginosa*, all exhibited a similar inhibitory effect, with most MICs around 20% (w/v) (Figure 2A). The Comvita Manuka 5+ had an MIC range of 15% (w/v) to 21% (w/v). The Comvita Manuka 10+ had an MIC range of 21% (w/v) to 27% (w/v) and the Comvita Manuka 15+ MIC ranged from 21% (w/v) to 33% (w/v). Two of the MDR clinical isolates tested had the lowest MIC for Manuka 5+, being <9% (w/v). This was also the same for one of those MDR isolates against Manuka 10+ and Manuka 15+, with MICs of 15% (w/v) and 21% (w/w), respectively. One of the non-MDR clinical isolates exhibited the highest MIC of all isolates tested, this was 27% (w/v), 27% (w/v) and 33% (w/v) for Manuka 5+, Manuka 10+ and Manuka 15+, respectively [31]. The other Manuka honeys tested had an MIC range of 9.5% (w/v) [34] to 17.5% (w/v) [32] against *P. aeruginosa* ATCC 27853, and an MBC range of 12.5% (w/v) [34] and 22.5% (w/v) [32]. The unclassified Manuka honey had the lowest MIC with the Manuka 10+ Kordel being the highest. 

The medical-grade honey 1 had the same MIC and MBC of 20% (w/v) for all *P. aeruginosa* isolates tested. The medical-grade honey 2 had the highest MIC and MBC of all honeys at 40% (w/v) for all isolates apart from one (Figure 2B) [37]. Revamil honey also had an MIC of 20% (v/v) [38], with the Activon medical-grade honey exhibiting the next best MIC of 12% (w/v) and MBC of 16% (w/v) [44]. The most effective honeys against *P. aeruginosa* were Surgihoney 1, 2 and 3 with MICs of 1.6% (w/v), 0.4% (w/v) and 0.1% (w/v), respectively. As well as MBCs of 1.6% (w/v) for Surgihoney 1, 1.6% (w/v) for Surgihoney 2 and 0.4% (w/v) for Surgihoney 3. The same study also used a Medihoney, which was less effective, with an MIC of 12% (w/v) and an MBC of >25.6% [39]. The medical-grade honey was the most consistent with an average of 10% MIC [46].

### 2.3. The Impact of Honey on Escherichia coli

**Figure 3:**
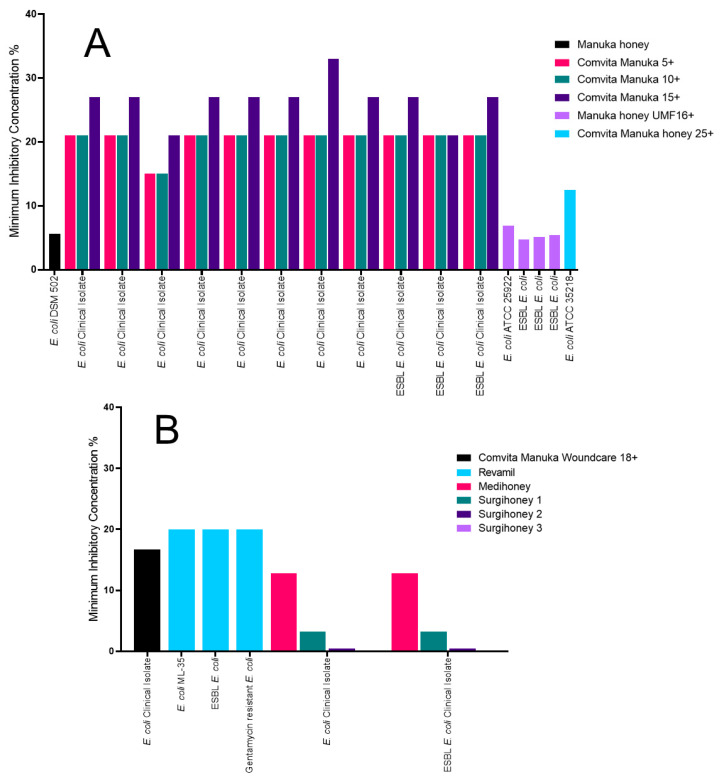
MICs of the *E. coli* isolates tested against a variety of Manuka and medical-grade honey. (**A**) All the Manuka honey MIC data used in this review, identifying an ungraded Manuka honey and Manuka honey UMF16+ as the most effective; and (**B**) all the medical-grade honey MIC data used in this review, identifying the three Surgihoneys as the most effective.

A range of inhibition was observed for *Escherichia coli* against a variety of Manuka honeys. Both the Manuka honey and Manuka honey UMF16+ were the most effective, having MICs of around 5% (v/v) (Figure 3A) [33,36]. The Manuka honey UMF16+ also had MBCs around 6% (v/v) [36]. The Manuka 25+ was not as effective and had an MIC and MBC of 12.5% (v/v) [35]. The Comvita Manuka 5+ and Comvita Manuka 10+ had the same inhibitory effect against all the *E. coli* isolates tested. One undefined clinical isolate tested was more susceptible and had an MIC of 15% (w/v) for both honeys. All other isolates tested had an MIC of 21% (w/v) for both honeys. The Comvita Manuka 15+ had a range of MICs, with two isolates having an MIC of 21% (w/v), one isolate of 33% (w/v) and all other isolates inhibited at 27% (w/v) [31].

The medical-grade honeys also had variation in inhibition for the *E. coli* isolates tested. The Revamil honey was the least effective, inhibiting all isolates tested at 20% (v/v) (Figure 3B) [38]. The Comvita Manuka Woundcare 18+ had improved efficacy, with an MIC of 16.7% (w/v) [42]. The most effective honeys against *E. coli* were Surgihoney 1, 2 and 3 with MICs of 3.2% (w/v), 0.4% (w/v) and 0.1% (w/v), respectively. They also had MBCs of 6.4% (w/v) for Surgihoney 1, 0.4% (w/v) for Surgihoney 2 and 0.2% (w/v) for Surgihoney 3 [39]. 

### 2.4. The Impact of Honey on Klebsiella pneumoniae

**Figure 4:**
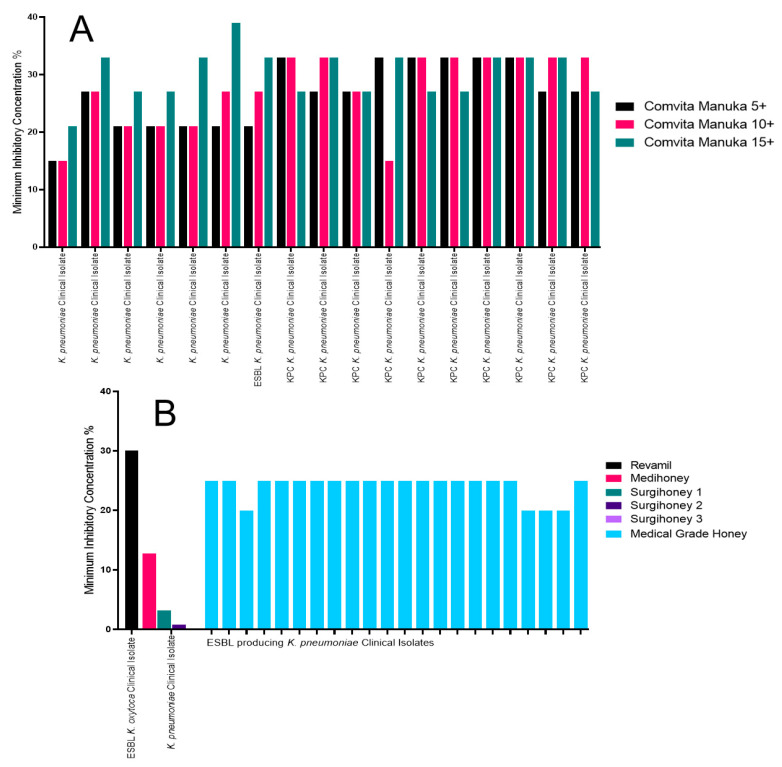
MICs of the *K. pneumoniae* isolates tested against a variety of Manuka and medical-grade honey. (**A**) The Manuka honey MIC data used in this review, identifying a similar response in MIC for all isolates; and (**B**) the medical-grade honey MIC data used in this review, identifying the three Surgihoneys as the most effective.

The three Manuka honeys tested against the *Klebsiella pneumoniae* clinical isolates displayed a range of MICs (Figure 4A). The Manuka 5+ had an MIC range of 15% (w/v) to 33% (w/v). Only one isolate had an MIC of 15% (w/v); however, 31.25% of the isolates had an MIC of 21% (w/v), another 31.25% of isolates had an MIC of 27% (w/v) and a further 37.5% of isolates had an MIC of 33%. All six of the isolates, with an MIC of 33% (w/v), were bla_KPC_ carbapenemase producers. The Comvita Manuka 10+ also exhibited a range of inhibition with MICs ranging from 15% (w/v) to 33% (w/v). The isolates with an MIC of 33% were all bla_KPC_ carbapenemase producers or a carbapenem-resistant but carbapenemase-negative isolate. The Comvita Manuka 15+ had an MIC range of 21% (w/v) to 39% (w/v). The isolate with an MIC of 21% (w/v) for the Comvita Manuka 15+ also had the lowest MIC of the other honeys tested. Only one isolate had an MIC of 39% (w/v) when tested against the Comvita Manuka 15+ [31].

The Revamil honey had the highest MIC at 30% (v/v) for the medical-grade honeys (Figure 4B) [38]. The medical-grade honey tested against a variety of ESBL-producing *K. pneumoniae* had an average MIC of 25%, with four isolates having an MIC of 20% [46]. 

### 2.5. The Impact of Honey on a Variety of Staphylococcus spp.

**Figure 5:**
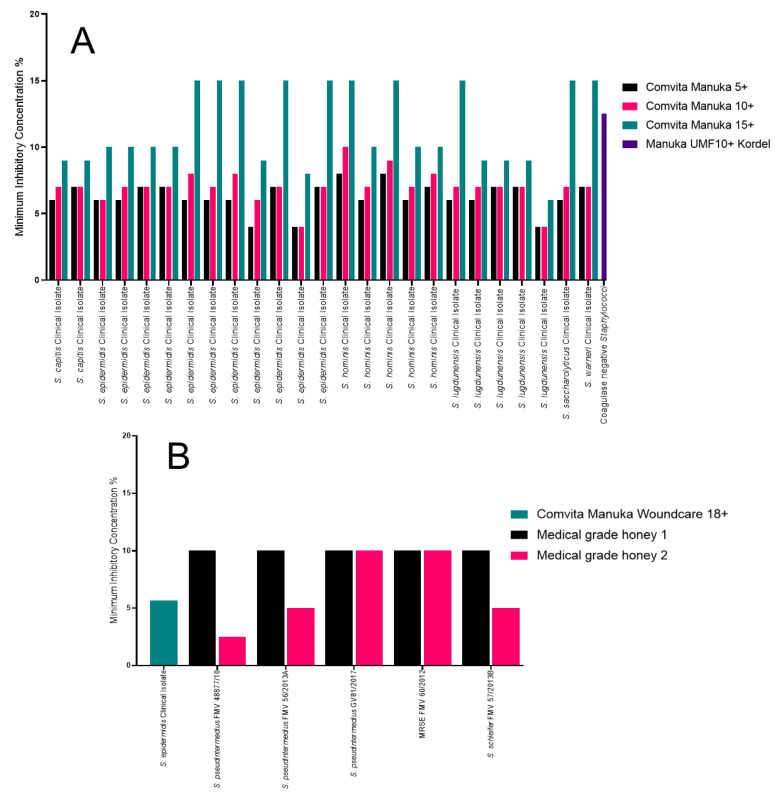
MICs of all other *Staphylococcus* isolates tested against a variety of Manuka and medical-grade honey. (**A**) All the Manuka honey MIC data used in this review, identifying the Comvita Manuka 5+ as the most effective honey; (**B**) all the medical-grade honey MIC data used in this review, identifying the Comvita Manuka Woundcare 18+ as the most effective.

A variety of other *Staphylococcus* strains were tested against different Manuka and medical-grade honeys (Figure 5A). The Comvita Manuka 5+ and Comvita Manuka 10+ had the lowest MICs ranging from 6% to 8% (w/v), with two *Staphylococcus epidermidis* and one *Staphylococcus lugdunensis* having an MIC of 5% (w/v). The Comvita Manuka 15+ MIC ranged from 9%–15% (w/v), with one *S. epiderimidis* isolate having an MIC of 8% (w/v); this was the same isolate with the lower MIC for Manuka 5+ and Manuka 10+ [31]. The Manuka UMF 10+ Kordel had the highest MIC at 12.5% (w/v) for a coagulase-negative staphylococci, and an MBC of >25% (w/v) [32]. 

The medical-grade honey 1 had an MIC of 10% (w/v), regardless of the *Staphylococcus* isolate tested (Figure 5B). The medical-grade honey 2 had more variation in MIC. The *Staphylococcus pseudintermedius* FMV 48877/10 had an MIC of 2.5% (w/v) and an MBC of 20% (w/v). Two isolates, *S. pseudintermedius* FMV 56/2013A and *Staphylococcus schleifer* FMV 57/2013B, had an MIC of 5% (w/v) and an MBC of 10% (w/v) and *S. pseudintermedius* GV81/2017 and MRSE FMV 60/2012 had an MIC of 10% (w/v) but an MBC of 20% (w/v) and 10% (w/v), respectively [37]. The Comvita Manuka Woundcare 18+ had an MIC of 5.67% (w/v) against the *S. epidermidis* clinical isolate tested [42]. 

### 2.6. The Impact of Honey on a Variety of Other Enterobacteriaceae

**Figure 6:**
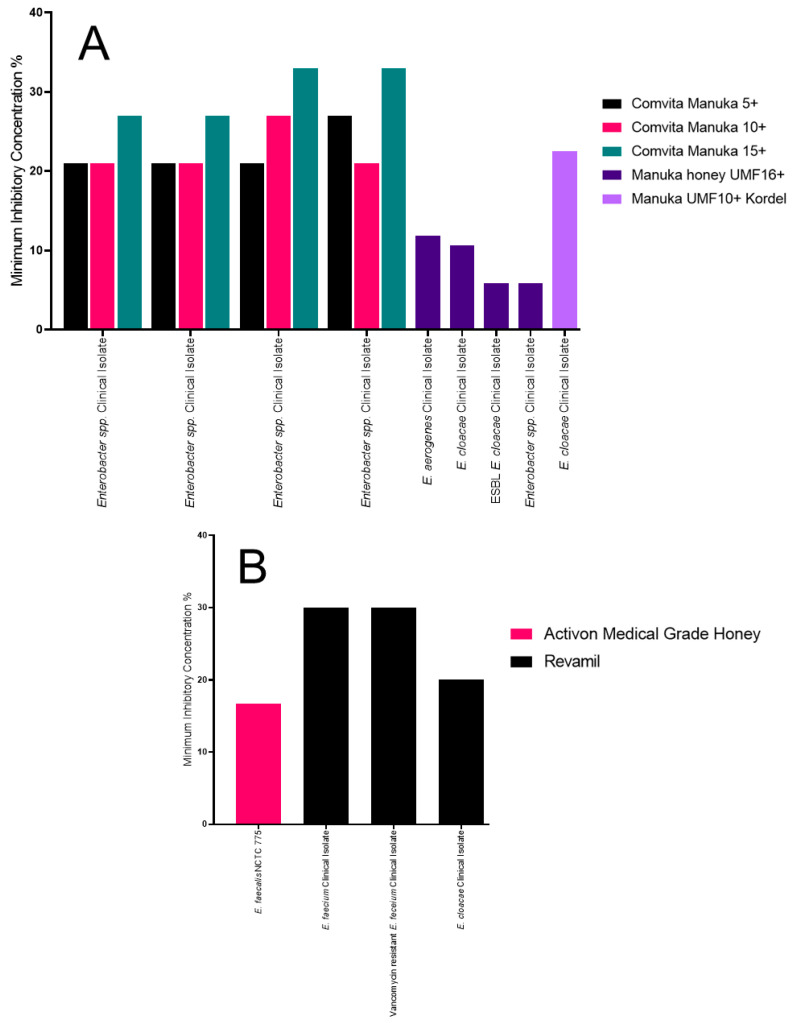
MICs of all other enteric bacterial isolates tested against a variety of Manuka and medical-grade honey. (**A**) All the Manuka honey MIC data used in this review, identifying the Manuka honey UMF16+ as the most effective; and (**B**) the medical-grade honey MIC data used in this review, identifying a similar activity between the Revamil and Activon medical-grade honeys.

The Manuka honey UMF16+ had the lowest MIC of all the enteric bacteria tested (Figure 6A) [36]. Both the ESBL *Enterobacter cloacae* and *Enterobacter* spp. had an MIC of 5.88% (v/v) but had MBCs of 6.75% (v/v) and 5.88% (v/v), respectively. *E. cloacae* had an MIC of 10.65% (v/v) and MBC of 16.6% (v/v) and *Enterobacter aerogenes* had an MIC of 11.89% (v/v) and an MBC of 16.6% (v/v). The Manuka UMF 10+ Kordel had an MIC of 22.5% (w/v) for the *E. cloacae* clinical isolate tested and an MBC of >25% (w/v) [32]. The Comvita Manuka 5+ had MICs of 21% (w/v) for three *Enterobacter* spp. and an MIC of 27% (w/v) for one isolate. The Comvita Manuka 10+ also had an MIC of 21% (w/v) for three of the isolates and an MIC of 27% (w/v) for one of the isolates. The higher MIC observed was for two different clinical isolates. The Comvita Manuka 15+ had an MIC of 27% (w/v) for two isolates and 33% (w/v) for the other two isolates [31].

The Revamil honey had an MIC of 30% (v/v) for both *Enterobacter faecium* and the vancomycin-resistant *E. faecium* clinical isolates tested (Figure 6B). The MIC was lower for *E. cloacae* at 20% (v/v) [38]. The Activon medical-grade honey had an MIC of 16.7% (w/v) for *E. faecalis* NCTC 775 [45].

### 2.7. The Impact of Honey on all Other Organisms Identified

**Figure 7:**
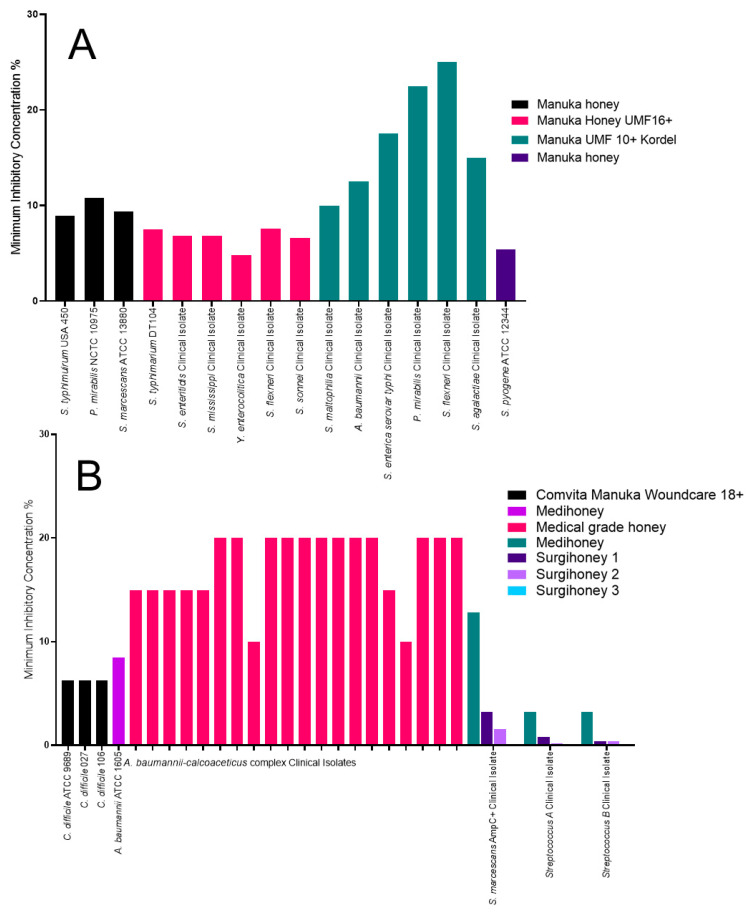
MICs of all other isolates tested against a variety of Manuka and medical-grade honey. (**A**) All the Manuka honey MIC data used in this review, identifying the ungraded Manuka honey and Manuka honey UMF16+ as the most effective; and (**B**) all the medical-grade honey MIC data used in this review, identifying a range of effectiveness between the honeys.

A range of other organisms were also tested against various Manuka and medical-grade honeys (Figure 7A). The Manuka UMF 10+ Kordel had a range of inhibition depending on the isolate tested. *Stenotrophomonas maltophilia* had the lowest MIC at 10% (w/v) and an MBC of 11.25% (w/v), whereas *Shigella flexneri* had the highest MIC at 25% (w/v) and an MBC of >25% (w/v). The other organisms tested were *Acinetobacter baumannii*, *Streptococcus agalactiae*, *Salmonella enterica serovar typhi* and *Proteus mirabilis*, with MICs of 12.5% (w/v), 17.5% (w/v) and 22.5% (w/v) and MBCs of 12.5% (w/v), 22.5% (w/v) and 17.5% (w/v), respectively [32]. An ungraded Manuka honey was tested against four different organisms, *Salmonella typhimuirum* USA 450, *Proteus mirabilis* NCTC 10975, *Serratia marcescans* ATCC 13880 and *Streptococcus pyogene* ATCC 12344, with MICs of 8.9% (v/v), 10.8% (v/v), 9.4% (v/v) and 5.4% (v/v), respectively [33]. The Manuka honey UMF16+ was tested against six different organisms, all of which had a similar MIC. *S. flexneri* had the highest MIC at 7.58% (v/v) but an MBC of 8.5% (v/v); the lowest MIC and MBC was for *Yersinia enterocolitica* at 4.79% (v/v) and 5.45% (v/v), respectively. The other organisms tested were *S. typhimirum* DT104, *Salmonella enteritidis*, *Salmonella mississippi* and *Shigella sonnei*, with MICs of 7.48% (v/v), 6.8% (v/v), 6.8% (v/v) and 6.61% (v/v) and MBCs of 10.65% (v/v), 8.5% (v/v), 8.5% (v/v) and 8.5% (v/v), respectively [36]. 

A range of *Acinetobacter baumannii* complex clinical isolates from burn patients were tested against a medical-grade honey (Figure 7B). Of these isolates, two had an MIC of 10%, five had an MIC of 15% and 12 had an MIC of 20% [46]. In a different study, *A. baumannii* ATCC 1605 had an MIC of 8.5% when tested against Medihoney [40]. Another study, comparing Medihoney and three Surgihoneys, identified that the Medihoney was the least effective, with MICs of 12.8% (w/v), 3.2% (w/v) and 3.2% (w/v) for *Serratia marcescans* AmpC producer, *Streptococcus* A and *Streptococcus* B, respectively. This was also observed for the MBCs, with *Serratia marcescans* AmpC producer having an MBC of >25.6% (w/v), and both *Streptococcus* A and *Streptococcus* B having MBCs of 12.8% (w/v). The Surgihoneys were more effective, with *S. marcescans* AmpC producer having MICs of 3.2% (w/v), 1.6% (w/v) and 0.1% (w/v) for Surgihoney 1, 2 and 3, respectively. *Streptococcus* A had MICs of 0.8% (w/v), 0.2% (w/v) and 0.03% (w/v) and *Streptococcus* B had MICs of 0.4% (w/v), 0.4% (w/v) and 0.03% (w/v) for Surgihoney 1, 2 and 3, respectively. This was also observed for the MBCs with *S. marcescans* AmpC producer having MBCs of 6.4% (w/v), 6.4% (w/v) and 0.2% (w/v) for Surgihoney 1, 2 and 3, respectively. Both *Streptococcus* A and *Streptococcus* B had MBCs of 1.6% (w/v) for Surgihoney 1 and 0.4% (w/v) for Surgihoney 2. For Surgihoney 3, *Streptococcus* A had an MBC of 0.1% (w/v) and *Streptococcus* B had an MBC of 0.05% (w/v) [39]. The other medical-grade honey tested was Comvita Manuka Woundcare 18+ against *Clostridium difficile* isolates with MICs and MBCs of 6.25% (v/v) for all three isolates [43]. 

### 2.8. Bacterial Pathogen Overall Susceptibility

The overall susceptibility of each bacterial pathogen varies, depending on the honey tested (Table 2). Overall, the most effective Manuka honey identified was Comvita Manuka 5+ and the ungraded Manuka honeys, having the lowest MICs for the majority of microorganisms. The least effective Manuka honey was Comvita Manuka 15+, having the highest MIC for the majority of microorganisms tested. Conversely, the most effective medical-grade honey identified was Surgihoney 3, having the lowest MICs observed overall. The least effective medical-grade honey was Revamil, having the highest MICs observed for the organisms tested. 

### 2.9. Bacterial Resistance Does Not Impact the Efficacy of Honey

The antibiotic resistance of bacterial pathogens was considered by some of the studies included in this review. The resistance profiles observed did not correlate with the MIC of the honey as expected (Table 3). Organisms with resistance to multiple antibiotics would be expected to have higher MICs when treated with either Manuka or medical-grade honey; this was not observed. One *P. aeruginosa* isolate had resistance to all antibiotics tested and an MIC of 10% when treated with medical-grade honey. Another *P. aeruginosa* isolate from the same study was only resistant to ampicillin/sulbactam but had an MIC of 15% when treated with the medical-grade honey [46]. This was also observed in two different studies with *P. aeruginosa*, *E. coli* and *E. cloacae* [31,36]. 

## 3. Discussion

Honey has been extensively studied for its antimicrobial activity, especially Manuka honey and medical-grade honey. A variety of medical-grade honeys are currently available, some of which are based on Manuka honey and others that have a different botanical source. Manuka-based medical-grade honeys are active due to the presence of methylglyoxal [19]. Non-Manuka-based medical-grade honeys utilise other antimicrobial components, such as bee defensin-1 in Revamil honey, or activity of reactive oxygen species in Surgihoney [47,48]. Regardless of the main antimicrobial component, the medical-grade honeys are able to inhibit a range of microorganisms. However, some are more effective than others, suggesting that the different antimicrobials within the honey can influence its effectiveness. 

Within a medical setting, honey is often used on chronic wounds and burns to prevent infection and aid healing due to its anti-inflammatory and antimicrobial activity [4]. It can be applied as a topical treatment or laced into a dressing [49,50]. As a topical ointment, honey provides a physical barrier between the wound and the environment whilst stimulating an inflammatory response and preventing against bacterial infection [51]. Dressings that are laced with honey can be changed less frequently than those without, resulting in less pain and discomfort for patients [52]. The use of honey in this way has been shown to reduce healing time, prevent scarring and protect from microbial infection, providing an effective treatment [2]. 

### 3.1. Manuka Honey

A variety of microorganisms were tested against three Comvita Manuka honeys of varying UMF (unique Manuka factor) grade; these were UMF5+, UMF10+ and UMF15+. A range of inhibition was observed for all of the microorganisms tested. The overall most effective honey identified by the study was Comvita Manuka UMF5+, inhibiting all microorganisms tested with the lowest MIC. Interestingly, the Comvita Manuka UMF10+ and UMF15+ were less effective, with the Manuka UMF15+ often having the highest MIC of all three honeys, regardless of the microorganism tested [31]. A different study also used Comvita Manuka honey but of a higher grade, UMF25+, and identified the same MIC, 12.5% (v/v), for MRSA and the clinical isolates of *P. aeruginosa* and *E. coli* [35]. Arguably, the Comvita Manuka UMF25+ should have been the most effective against the isolates tested because of it having the highest UMF grade. Therefore, it should have the highest amount of MGO and consequently the most antimicrobial activity. However, this was not the case with regard to *S. aureus*. Both the Comvita Manuka 5+ and 10+ had lower MICs than the Comvita Manuka UMF25+ and 20 out of 48 *S. aureus* isolates tested had a lower MIC for Comvita Manuka 15+. This was also observed for the Manuka 10+ Kordel and Comvita Manuka 25+, where both MICs were the same [32,35]. The Comvita Manuka 25+ did, however, have a lower MIC for both *E. coli* and *P. aeruginosa* when compared to any of the Comvita Manuka 5+, 10+ and 15+, as well as the Manuka 10+ Kordel. One explanation for this could be in the mechanism of action of methylglyoxal (MGO), the main contributing factor to the Manuka honey’s antimicrobial activity. Observations using scanning electron microscopy and transmission electron microscopy identified that, in the presence of MGO, both *E. coli* and *B. subtilis* had loss of flagella and fimbriae as well as displaying shrinking and rounding of the cell [53]. *S. aureus* does not have flagella or fimbriae and is coccidal in shape, rather than a rod. This indicates that MGO would not be exerting this effect on *S. aureus* due to the absence of these features. Moreover, the presence of MGO has been suggested to have little effect on *S. aureus* other than the result of an increase in cells with fully formed septa and no other structural changes [54]. In both studies using the Comvita Manuka honey, other contributing factors were not taken into account, such as hydrogen peroxide or bee defensin-1. Hydrogen peroxide and bee defensin-1 are present in all honey samples at varying levels. These components both have different mechanisms of action. Hydrogen peroxide causes DNA damage and, although the action of bee defensin-1 has not been fully elucidated, other defensin proteins disrupt the cell membranes [55,56], suggesting that these, or a currently unknown component of Manuka honey, causes the antimicrobial affect against *S. aureus*. Therefore, it could be suggested that MGO does not directly impact *S. aureus*, resulting in the UMF classification being irrelevant to this pathogen. However, the UMF rating would be relevant to other pathogens, such as *E. coli*, *P. aeruginosa* and *B. subtilis*, due to the lower MIC observed for the higher-grade honey. 

The broth microdilution method was used by all three of these studies. However, Tan et al. [32] used a 48-well microtiter plate, Sherlock et al. [35] used a 96-well plate and Girma et al. [31] did not state. This difference in method could have impacted the results between the studies. The study conducted by Tan et al. [32] used 0.5 mL of honey at the desired concentration with 0.5 mL of bacterial culture adjusted to 5 x 10^5^ CFU/mL, whereas the study by Sherlock et al. [35] used 190 µL of honey at the desired concentration and 10 µL of bacterial culture adjusted to a 0.5 McFarland standard. Girma et al. [31] did not state the volumes used but had a final working volume of 0.1 mL inoculated with bacterial culture of 5 × 10^4^ CFU/mL. Additionally, Sherlock et al. [35] had a more defined methodology, which took into account an initial T0 reading that was subsequently subtracted from the T24 reading to provide a more accurate reading. The method used by Tan et al. [32] did not consider this and also noted two different MICs, one from a visual inspection and one spectrophotometric. For the purpose of the analysis of this review, the visual inspection was disregarded, and the MIC observed for the spectrophotometric data was used. Girma et al. [31] did not state the method used to determine the MIC. 

Both Sherlock et al. [35] and Tan et al. [32] did observe the minimum bactericidal activity (MBC) of the honey. Interestingly, the Comvita Manuka 25+ had the same MIC and MBC; however, the Manuka 10+ Kordel had an MBC reported of 10% higher than the MIC. This difference in MIC and MBC between the two honeys could demonstrate the difference in Manuka honey rating, identifying the Comvita Manuka 25+ has better bactericidal activity than the Manuka 10+ Kordel. The hydrogen peroxide content was considered in both of these studies; however, the content of hydrogen peroxide within the honey samples was not measured. By measuring the hydrogen peroxide content, the assay to remove it could be performed and the honey retested to observe the amount of hydrogen peroxide removed from the honey. Additionally, different methods were used by both studies to observe the effect of hydrogen peroxide removal. Regardless of this, both studies identified that the removal of hydrogen peroxide had little to no impact on the antimicrobial activity, further supporting the overriding evidence that the increased activity of Manuka honey is attributed to the presence of MGO. In the study conducted by Lin et al. [36], the hydrogen peroxide content was also considered and an assay developed to remove it and retest the activity of the honey. For *S. tiphymurium* phage type 4, *S. tiphymurium* DT104 and *S. enteritidis* the MIC did not change upon hydrogen peroxide removal. However, all other isolates tested did have an increase in MIC, although this was not significant and was still lower than compared to an artificial honey.

With regard to Manuka honey, overall, the Gram-positive clinical isolates had lower MICs than the Gram-negative clinical isolates. It has been suggested previously that honey is more effective against Gram-positive microorganisms than Gram-negative ones [12,13,14]. However, this is not strictly the case. The study conducted by Tan et al. [32] identified a range of MICs for a variety of organisms tested against Kordel’s Manuka honey UMF10+. The Gram-positive organisms included in the study were *S. aureus*, coagulase-negative staphylococci and *S. agalactiae* with MICs of 12.5% (w/v), 12.5% (w/v) and 15% (w/v), respectively. The lowest MIC observed, 10% (w/v), was for Gram-negative *S. maltophilia* and the MIC for *A. baumannii* (12.5%) matched that of *S. aureus*. Other Gram-negative organisms did, however, have higher MICs, and the highest observed was 25% for *S. flexneri*. In the study conducted by Lin et al. [36], a variety of Gram-negative organisms were treated with Manuka honey 16+ and MICs as low as 4.72% (v/v) for an ESBL *E. coli* were observed, with 11.89% (v/v) for *E. aerogenes* being the highest. Comparing these values to those observed for *S. aureus* and the other staphylococci treated with Comvita Manuka 15+ [31], the MICs for the Gram-negative ones are significantly lower [36]. Furthermore, in the study conducted by Sherlock et al. [35], the same MIC was observed for *S. aureus*, *P. aeruginosa* and *E. coli* when treated with Manuka honey UMF25+, suggesting that Manuka honey is not more effective against Gram-positive microorganisms than the Gram-negative ones. Nevertheless, this demonstrates that Manuka honey has a broad spectrum of activity. 

Considering the classification of Manuka honey, those with higher UMF grades would be considered the most effective. However, this was not observed. The first observation of this is for the Comvita Manuka UMF5+, 10+ and 15+, where the most effective honey was the UMF5+. Another observation of this is with an ungraded Manuka honey having the lowest MIC observed for all of the *S. aureus* isolates at 2.7% (v/v) [33]. However, this was only tested against one isolate and does not provide an accurate presiding picture of the honey’s activity. A screening of this honey against a range of *S. aureus* isolates could have provided a better overall picture of the activity of this honey. Nevertheless, it identifies the potent activity that Manuka honey possesses. Additionally, the Manuka 16+ had lower MICs than the Comvita Manuka 25+ when tested against various *E. coli* [35,36]; also, an ungraded Manuka honey [34] had a lower MIC for *P. aeruginosa* than the Manuka 10+ Kordel and Comvita Manuka 25+ [32,35]. However, this was also a lone example and so does not provide a detailed example. However, a variety of factors could be implicated in these discrepancies, including the final MGO concentration at the time of testing and the hydrogen peroxide content. These factors were not assessed by most authors and are therefore largely unknown. However, during the Manuka honey ripening process, dihydroxyacetone (DHA) is converted into MGO. Girma et al. [31] suggested that during the time of manufacturing there could have been higher DHA and lower MGO, resulting in the lower classification of the Manuka 5+; this indicates that, at the time of testing the antibacterial activity, higher MGO could have been present. This is possible, but not the only factor. One main component of all honeys is hydrogen peroxide. Levels of hydrogen peroxide can vary between honey samples and a higher level could have been present in the Manuka 5+, exerting an increased antibacterial effect than the Manuka 10+ or 15+. The presence of bee defensin-1 and other polyphenolic compounds are also not considered, both of which can impact the antibacterial activity of honey through direct mechanisms or oxidation activation of hydrogen peroxide [57,58]. Furthermore, other factors, such as storage condition and sample preparation, could impact the antimicrobial activity of the honey [59]. Therefore, where possible, other factors should also be considered and measured to provide a better insight into the antimicrobial activity of honey. 

The MDR status of all microorganisms within this review have not been provided; however, they were for some. The study conducted by Girma et al. [31] included *K. pneumoniae* bla_KPC_ and carbapenemase producers, as well as a carbapenem-resistant but carbepenemase-negative strain; ESBL-producing *E. coli*; and MDR *P. aeruginosa*. Although the drug resistance profiles were not provided, the authors did state that the MDR status was determined. For the *E. coli* and *P. aeruginosa* clinical isolates, the drug resistance status of the isolates did not appear to affect the efficacy of the honey. However, with regard to the *K. pnauemoniae* clinical isolates, it appeared that the bla_KPC_ carbapenemase producers had higher MICs for Manuka 5+ and Manuka 10+. This was not observed for the Manuka 15+, suggesting that, depending on the organism, MDR status may not be important when considering treatment with Manuka honey. If this is the case, it could be an important finding for the growing concern of antimicrobial resistance and infections caused by multidrug-resistant organisms. In another study by Lin et al. [36], three ESBL *E. coli*, one ESBL *E. cloacae* and one ESBL *Enterobacter* spp. had their resistance profiles determined. All five of these isolates were resistant to amoxicillin, amox/clauvulanate and cefaclor, among many other drugs such as trimethoprim, ciprofloxacin, cefuroxime and gentamycin. When treated with Manuka honey UMF16+, all five of these organisms had a lower MIC than the *E. coli* ATCC 25923 and other *E. cloacae* tested (Table 3). Furthermore, the ESBL-producing isolates had the lowest MIC observed for all isolates tested, apart from *Y. enterocolitica* [36]. This further suggests that the drug-resistant status of the bacteria does not impact the efficacy of the honey and that Manuka honey could be an effective treatment, especially against MDR strains. Ultimately, Manuka honey could be used to combat infections caused by multidrug-resistant organisms, an important step in the fight against antimicrobial resistance. 

### 3.2. Medical-Grade Honey

By comparison, the medical-grade honeys had larger variation in activity when compared to the Manuka honeys. The MIC ranged between 0.01% for Surgihoney 3 and up to 20% for Revamil when tested against the MRSA clinical isolates [38,39]. The variations observed could be due to honey origin, as not all medical-grade honey is based on Manuka honey. Revamil honey is produced in greenhouses under standardised conditions [60]; the manufacturer has not shared the floral source, however they state it is not based on Manuka honey. Similarly, more than one type of Surgihoney is marketed and the floral source is also not shared. In the absence of this data, it is difficult to determine why these honeys are more or less effective than each other. Nevertheless, it is important to determine which medical-grade honeys are the most effective against each organism so the best treatment options can be provided. 

The most effective honeys observed were Surgihoneys 1, 2 and 3, with MICs averaging 3.2%, 0.8% and 0.1%, respectively [39]. Surgihoney, now called SurgihoneyRO, is not based on a specific botanical source and is sourced from several locations. The antimicrobial activity has been enhanced to produce elevated levels of reactive oxygen species, resulting in it being defined as an engineered honey [61]. The higher the grade of Surgihoney, the more enhanced the activity. Therefore, Surgihoney 1 was not as effective as Surgihoney 2 and subsequently Surgihoney 3 was the most effective. 

The medical-grade honey that was tested against a variety of MRSA, *P. aeruginosa*, *K. pneumoniae* and *A. baumannii–A. calcoaceticus* (ABC) complex clinical isolates had an MIC range of 5%–25%, depending on the organism tested [46]. The MRSA isolates had the largest range in MIC, of 5%–20%. *P. aeruginosa* ranged between 10% and 15% and ABC ranged between 10% and 20%. The highest MICs observed were for *K. pneumoniae* between 20% and 25%. No further information was provided by the study about the honey that was used, other than it was a medical-grade honey and the weight-to-volume or volume-to-volume dilutions were not stated. Therefore, the possible active antimicrobial agent is unknown. Resistance profiles were provided for the isolates tested, and almost all of the clinical isolates were multidrug resistant. Interestingly, the resistance profiles did not correlate with the MIC of the honey as expected (Table 3). The organisms identified with resistance to multiple antibiotics often had the lowest MICs for the honey when compared to isolates with resistance to only one or two antibiotics. Due to the nature of honey, it is largely considered that there is not one singular mechanism that provides the antimicrobial activity. Since antibiotics have a specific target, such as disrupting cell membranes or DNA damage, bacteria can often gain resistance. When drugs are used in combination, a greater pressure is exerted on the bacteria and it is less likely to gain resistance. If honey is effective by utilising multiple mechanisms, this could explain the discrepancy between multidrug resistance profiles and the effect of the honey. 

Overall, the least effective honey was Revamil, often having the highest MIC for the isolates tested. Although the botanical source has not been shared, the antimicrobial activity of Revamil is attributed to hydrogen peroxide activity and bee defensin-1, not methylglyoxal [47]. This could be the reason the Revamil honey is the least effective of the medical-grade honeys. 

### 3.3. Clinical Application of Honey

Honey has been used as medicine for centuries, but since the licencing of honey-based products in the European Union in 2004, its use in the United Kingdom has been limited to topical treatments of acute and chronic wounds. Preparations include impregnated dressings, sheet dressings and wound gel [62]. 

Wounds are created by the disruption of the dermis and epidermis of the skin and subsequent infiltration of the wound with a mixture of commensal bacteria and potentially pathogenic bacteria from the environs. Bacterial presence in the wound has shown to be helpful initially as it causes inflammation and stimulates healing [63]. Typical acute colonisations may be attributed to aerobic Gram-positive cocci such as *Staphylococcus* and *Streptococcus* spp. Chronic wounds are contaminated with a broader range of aerobic and anaerobic bacteria [64], including bacteria we have identified in this review such as *P. aeruginosa* and *S. epidermis*. 

Chronic wounds are more difficult to treat, particularly exacerbated by antibiotic resistance, and with this comes significant healthcare costs, with the annual wound-care market expected to reach USD15–22 billion by 2024 [65]. In addition to antibiotic resistance, chronic wounds are challenging to treat due to the formation of bacterial biofilms. A biofilm is a matrix of bacteria and extracellular polymeric substances [66], which can delay the healing process [67]. The formation of a physical barrier restricts penetration of antibiotics and reduces the chance of wound disinfection [68]. Lu et al. (2014) showed that Manuka honey infiltrates the biofilm and kills bacterial cells, showing that honey with a higher UMF, and consequently MGO content, produced greater anti-biofilm activity [69]. Critically, they found administering MGO on its own does not have this effect, emphasising the importance that the other components play in honey’s bactericidal action. 

Honey demonstrates several characteristics that make it effective for wound management; it has both antimicrobial and anti-inflammatory properties, it promotes autolytic debridement, promotion of granulation [70] and reduces malodour [71]. In part, its antimicrobial activity is attributed to its physical properties, including a high osmotic pressure and low pH [72]. Additionally, honey’s antimicrobial activity is due to the presence of methylglyoxal (MGO), hydrogen peroxide, flavonoids and phenols, as well as the enzyme bee defensin-1 [5]. Interestingly, as we have identified, the concentration of MGO (and subsequent Unique Manuka Factor, UMF) does not necessarily correlate with the minimum inhibitory concentration (MIC) of certain bacteria, suggesting a multimodal mechanism of action. 

The most significant finding of our review is that the efficacy of honey is not affected by bacterial resistance. The increasing prevalence of multidrug-resistant (MDR) bacteria is a key concern effecting modern society. In the EU, antibiotic resistance causes 25,000 deaths per year and more than two million additional hospital days [73]. The identification of an agent that appears to have equal efficacy against non-MDR and MDR bacteria is a promising starting point for further investigation.

Of course, in vitro susceptibility of bacteria to antimicrobials does not necessarily translate into in vivo efficacy. We have identified that Comvita Manuka 5+ reliably demonstrated the lowest MIC for treating *S. aureus* with one study showing an MIC of 5–7%, irrespective of methicillin resistance [31]. Comvita Manuka 5+ had similarly low MICs for *S. epidermidis* (around 5%). However, for *E. coli*, the Surgihoneys were particularly effective, with UMF16+ being bactericidal against ESBL-producing bacteria [39]. 

These findings clearly demonstrate that a number of honeys are effective against MDR bacteria in vitro at low concentrations. This is encouraging as we know that some patient populations have difficult-to-treat infections caused by bacteria requiring high concentrations of antibiotics, such as intensive care patients [74,75] and immunocompromised patients [76]. Using honey to treat wound infections in these patients may allow a reduced dose or duration of antibiotics, improving both patient experience and therapeutic efficacy.

In vitro studies have identified a synergy between honey and certain antibiotics with MRSA [77,78]. Jenkins and Cooper (2012) found that the application of 5% Manuka honey reversed the resistance of MRSA to oxicillin and Muller et al. (2013) identified a synergism of honey with rifampicin. Conversely, Liu et al. (2018) have found that honey can act antagonistically with oxacillin, as well as being antagonistic with clindamycin and gentamicin, but also found a synergistic effect of honey with rifampicin [79]. These contradictory findings highlight the importance of a systematic data analysis incorporating multiple studies to draw conclusions of clinical significance. 

Whilst laboratory-based studies are promising, at present there is a shortage of clinical data to support the topical use of honey. Guidelines published by the National Institute for Health and Care Excellence [80] on chronic wounds, which has been heavily influenced by a Cochrane review by Jull et al. (2015) [50], have concluded that there is little good-quality clinical evidence to support the use of honey dressings for chronic wounds such as diabetic foot ulcers, pressure ulcers and infected wounds. The lack of evidence in this area has prompted NICE to call for further randomised trials regarding the use of antimicrobial dressings in treating chronic wounds [81]. 

Honey is currently used in clinical settings as a topical application for acute and chronic wounds and bacterial resistance to honey has not been identified [19]. Furthermore, honey’s multimodal mechanism of action and consequent efficacy against MDR bacteria combined with its anti-biofilm activity make it an exciting prospect for future research. In vitro evidence suggesting synergy with certain antibiotics and even reversal of resistance of antibiotics to *S. aureus* in combination with our review findings adds weight to the argument that topical honey administration could play an important role in wound management. Randomised clinical trials are required to explore further the impact of medical honeys against MDR and non-MDR organisms in the clinic, to determine if administration of honey can improve wound healing times and reduce the duration and dosing of antibiotic treatment. 

## 4. Methods

**Figure 8:**
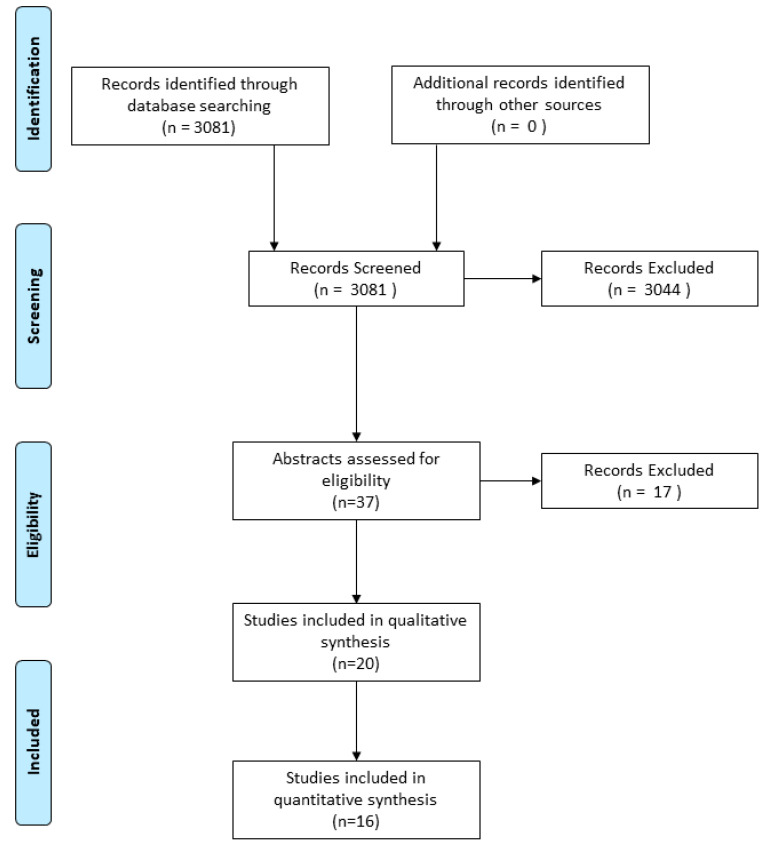
PRISMA diagram showing how records were screened and selected to be used for the systematic review.

To begin the systematic review, a search of the literature was conducted using the PubMed, ScienceDirect, Google Scholar and Web of Science databases by two independent researchers. The search criteria included combinations of keywords, including “Manuka honey”, “medical grade honey”, “Medihoney”, “multidrug resistant”, “multidrug resistance” and “multidrug resistant organism”. This produced a total of 3081 publications, of which 32 articles were selected based on the article type, title and abstract fulfilling the search criteria. Of these, 17 articles were removed due to not reporting antimicrobial activity of Manuka honey or medical-grade honey, duplicate papers identified by independent researchers or researchers being unable to access the article. This resulted in the inclusion of 20 papers for the systematic review to be conducted (Figure 8). 

The information extracted from the papers included the organisms tested, methods used, honey used and minimum inhibitory concentrations (MICs) recorded. Two main methods were used in the majority of the papers, broth dilution and the agar dilution assay. Therefore, the data were divided into these two categories. The data were recorded in Mircosoft Excel and organised in order from lowest to highest inoculum used. The data were further divided into Manuka honey and medical-grade honey. Manuka honey was defined as a honey originating from *Leptospermum* spp. and did not state any medical-grade classification. Medical-grade honey was defined as any honey stating a medical grade or medical use. The collected data were then processed in GraphPad Prism 8 for graphical summary. Due to the large set of data generated, the focus of the results was on the broth dilution method, using 16 of the selected papers. 

## 5. Conclusions

Both Manuka honey and medical-grade honeys are affective against a variety of microorganisms. The overall most effective honey identified in this review is Surgihoney, with MICs as low as 0.1%. However, some Manuka honeys can be just as effective as medical-grade honeys, depending on the organism it is being used against. Considering the Manuka honey, the ungraded Manuka honeys had lower MICs than those graded 15+ and 25+. Furthermore, the Manuka honeys are largely unregulated compared to the medical-grade honeys produced under controlled conditions. Each Manuka honey could be largely different to the next due to this, making it difficult to determine if the Manuka honey could be as effective for treatment as the medical-grade honeys. However, not all the medical-grade honeys are equally effective. The Revamil honey was identified as the least effective medical-grade honey and comparing MICs to the Manuka honey shows that, with regard to *S. aureus*, *E. coli* and *K. pneumoniae*, the Manuka honeys often had the lower MIC. With this in mind, Surgihoney should be considered as the most effective treatment option; however, clinical data alongside this would provide a stronger basis for recommendation. Manuka honey should not be discounted as a treatment option or basis for medical-grade honey, especially considering its effect on biofilms. Interestingly, one of the main findings from this review is that the drug-resistant status of the organisms tested did not appear to impact the efficacy of the honey. Remarkably, the multidrug-resistant organisms were often more susceptible to honey treatment. Therefore, the treatment of infections, especially those caused by MDR organisms, should be considered for honey therapy, potentially in combination with antibiotics. Furthermore, honey therapy could be an important strategy to combat infections and should be comprehensively trialled for use within a medical setting. 

## Figures and Tables

**Table 1:** Summary of the honey used in this review, the organisms tested and the antibiotic resistance of those organisms.

Honey	Manuka or Medical Grade	Organisms Tested	Antibiotic Resistance
Comvita Manuka 5+, Comvita Manuka 10+ and Comvita Manuka 15+ [31]	Manuka honey	*Staphylococcus aureus*, *Pseudomonas aeruginosa*, *Escherichia coli*, *Klebsiella pneumoniae*, *Staphylococcus epidermidis*, *Staphylococcus lugdunensis*, *Staphylococcus capitis*, *Staphylococcus hominis*, *Staphylococcus saccharolyticus*, *Staphylococcus warneri* and *Enterobacter* spp.	Methicillin resistant, multidrug resistant, carbapenamase producers, extended-spectrum β-lactamase producers.
Manuka honey UMF10+ Kordel [32]	Manuka honey	*S. aureus*, *P. aeruginosa*, Coagulase negative *Staphylococcus*, *Enterobacter cloacae*, *Stenotrophomonas maltophilia*, *Shigella flexneri*, *Acinetobacter baumannii*, *Streptococcus agalactiae*, *Salmonella enterica serovar typhi* and *Proteus mirabilis*	Methicillin resistance.
Ungraded Manuka honey [33]	Manuka honey	*S. aureus*, *P. aeruginosa*, *E. coli*, *S. typhimurium*, *P. mirabilis* and *Staphylococcus pyogenes*	N/A
Ungraded Manuka honey [34]	Manuka honey	*P. aeruginosa*	N/A
Comvita Manuka 25+ [35]	Manuka honey	*S. aureus*, *P. aeruginosa* and *E. coli*	Methicillin resistance and oxacillin resistance.
Manuka honey UMF 16+ [36]	Manuka honey	*E. coli*, *E. cloacae*, *Enterobacter* spp., *Enterobacter aerogenes*, *S. typhimurium*, *Salmonella enteritidis*, *Yersinia enterocolitica*, *S. flexneri* and *Shigella sonnei*	Extended spectrum β-lactamase producers.
Medical-grade honey 1 and Medical-grade honey 2 [37]	Medical-grade honey	*S. aureus*, *P. aeruginosa*, *Staphylococcus pseudintermedius*, *Staphylococcus* epidermidis and *Staphylococcus schleiferi*	Methicillin resistance.
Revamil [38]	Medical-grade honey	*S. aureus*, *P. aeruginosa*, *E. coli*, *Klebsiella oxytoca*, *Enterococcus faecium*, *E. cloacae* and *S. epidermidis*	Methicillin resistance, gentamycin resistance and extended spectrum β-lactamase producers.
Surgihoney 1, Surgihoney 2 and Surgihoney 3 [39]	Medical-grade honey	*S. aureus*, *P. aeruginosa*, *E. coli*, *K. pneumoniae*, *Sterptococcus A*, *Streptococcus B*, and *Serratia marcescans*	Methicillin resistance, vancomycin resistance and extended spectrum β-lactamase producers.
Medihoney [39,40]	Medical-grade honey	*S. aureus*, *A. baumannii*, *P. aeruginosa*, *E. coli*, *K. pneumoniae*, *Sterptococcus A*, *Streptococcus* and *Serratia marcescans*	Methicillin resistance, vancomycin resistance, extended spectrum β-lactamase producers, oxacillin resistance and multidrug resistance.
Comvita Manuka Woundcare 18+ [41,42,43]	Medical-grade honey	*S. aureus* and *P. aeruginosa*, *E. coli*, *S. epidermidis* and *Clostridium difficile.*	Methicillin resistance, clindamycin, moxifloxacin, fluoroquinolone and rifampicin resistance.
Activon medical-grade honey [44,45]	Medical-grade honey	*S. aureus*, *P. aeruginosa*, *E. coli* and *E. faecalis*	N/A
Medical-grade honey [46]	Medical-grade honey	*S. aureus*, *P. aeruginosa*, *E. coli*, *K. pneumoniae*, *E. faecalis* and *Acinetobacter baumannii–A. calcoaceticus* complex	Methicillin resistance and extended spectrum β-lactamase producers.

**Table 2:** Overall bacterial pathogen susceptibility: identifying the most effective and least effective Manuka and medical-grade honeys.

Bacterial Pathogen	Most Effective Honey		Least Effective Honey	
	Manuka	Medical Grade	Manuka	Medical Grade
*S. aureus*	Manuka honey ungraded, MIC of 2.7% [33]	Surgihoney 3, MIC of 0.01% [39]	Comvita Manuka 15+, MIC of 15% [31]	Activon medical-grade honey, MIC of 12% [45]
MRSA	Comvita Manuka 5+, MIC of <5% [31]	Surgihoney 3, MIC of 0.01% [39]	Comvita Manuka 15+, MIC of >15% [31]	Revamil honey, MIC of 20% [38]
*P. aeruginosa*	Manuka honey ungraded, MIC of 9.5% [34]	Surgihoney 3, MIC of 0.1% [39]	Comvita Manuka 15+, MIC of 33% [31]	Medical-grade honey 2, MIC of 40% [37]
MDR *P. aeruginosa*	Comvita Manuka 5+, MIC of <9% [31]	Surgihoney 3, MIC of 0.1% [39]	Comvita Manuka 15+, MIC of 27% [31]	N/A
*E. coli*	Manuka honey ungraded, MIC of 5.6% [33]	Surgihoney 3, MIC of 0.1% [39]	Comvita Manuka 15+, MIC of 33% [31]	Revamil honey, MIC of 20% [38]
ESBL producing *E. coli*	Manuka honey 16+, MIC of 5.08% [36]	Surgihoney 3, MIC of 0.1% [39]	Comvita Manuka 15+, MIC of 27% [31]	Revamil honey, MIC of 20% [38]
*K. pneumoniae*	Comvita Manuka 10+, Mic of 15% [31]	Surgihoney 3, MIC of 0.1% [39]	Comvita Manuka 15+, MIC of 39% [31]	Revamil honey, MIC of 30% [38]
*S. epidermidis*	Comvita Manuka 5+, MIC of <5% [31]	N/A	Comvita Manuka 15+, MIC of 15% [31]	N/A
*Enterobacter spp.*	Manuka honey UMF16+, MIC of 5.88% [36]	N/A	Comvita Manuka 15+, MIC of 33% [31]	N/A
*E. cloacae*	Manuka honey UMF16+, MIC of 5.88% [36]	N/A	Manuka honey UMF10+ Kordel, MIC of 21% [32]	N/A
*A. baumannii*	N/A	Medihoney, MIC of 8.5% [40]	N/A	Medical-grade honey, MIC of 20% [46]
*S. marcescans*	N/A	Surgihoney 3, MIC of 0.1% [39]	N/A	Medihoney, MIC of 12.8% [39]

**Table 3:** Examples of bacterial resistance and the efficacy of honey.

Organism	Antibiotic Resistance	Honey and MIC
MRSA [46]	Clindamycin, erythromycin, levofloxacin and moxifloxacin	Medical-grade honey, 5%
MRSA [46]	Erythromycin, levofloxacin and moxifloxacin	Medical-grade honey, 20%
MRSA [46]	Gentamycin, levofloxacin, tetracycline and trimethoprim	Medical-grade honey, 5%
MRSA [46]	Erythromycin	Medical-grade honey, 15%
*P. aeruginosa* [46]	Amikacin, gentamycin, tobramycin, ampicillin/sulbactam, cefepime, ceftazidime, pipericillin/tazobactam, levofloxacin, ciprofloxacin, imipenem and meropenem	Medical-grade honey, 10%
*P. aeruginosa* [46]	Ampicillin/sulbactam	Medical-grade honey, 15%
ABC complex [46]	Amikacin, gentamycin, tobramycin, ampicillin/sulbactam, cefepime, ceftazidime, pipericillin/tazobactam, levofloxacin, ciprofloxacin and meropenem	Medical-grade honey, 10%
ABC complex [46]	Pipericillin/tazobactam	Medical-grade honey, 20%
Multidrug-resistant *P. aeruginosa* [31]	Not Specified	Comvita Manuka 5+, <9% Comvita Manuka 10+, 15% Comvita Manuka 15+, 21%
*P. aeruginosa* [31]	No resistance	Comvita Manuka 5+, 27% Comvita Manuka 10+, 27% Comvita Manuka 15+, 33%
*E. coli* [36]	Amoxycillin, Amoxy/Clavulanate, Cefaclor, Trimethoprim, Ceftriaxone, Cefuroxime, Ceprofloxacin, Cotrimoxazole and Gentamicin	Manuka honey UMF16+, 5.08%
*E. coli* ATCC 25923 [36]	Not Specified	Manuka honey UMF16+, 6.87%
*E. cloacae* [36]	Amoxycillin, Amoxy/Clavulanate, Cefaclor	Manuka honey UMF16+, 5.88%
*E. cloacae* [36]	Not Specified	Manuka honey UMF16+, 10.65%
